# Effectiveness of an intervention in multicomponent exercise in primary care to improve frailty parameters in patients over 70 years of age (MEFAP-project), a randomised clinical trial: rationale and study design

**DOI:** 10.1186/s12877-018-1024-8

**Published:** 2019-01-28

**Authors:** M. V. Castell, A. Gutiérrez-Misis, M. Sánchez-Martínez, M. A. Prieto, B. Moreno, S. Nuñez, R. Triano, M. P. de Antonio, C. Mateo, M. D. Cano, A. Garrido, R. Julian, E. Polentinos, R. Rodriguez-Barrientos, A. Otero Puime, MEFAP Group, Virtudes Enguita Perez, Belen de la Fuente Martin, Raquel Carcerén y Murciano, Rafael Ruiz Morote Aragón, Mª. Victoria Diaz Puente, Elena Fernández García, Enma González Nespereira, Maria Luisa Asensio Ruiz, Elena Orio Moreno, Daniel Pascual Diez, Mercedes Piñeiro Rodriguez, Susana López Cotón, Eva María Illana, Maria Teresa Blanco Ramos, Maria Concepción Hernández de la Luna, Catalina Mª. Santiago Gonzalez, Isabel Contreras Calzada, David Morales Tereja, Ana Maria Rosas Hernandez, Marta Agüero Martin, Verónica Andrés Sanz, Ricardo Duque Heras, Beatriz Carmen Durán Fernández, Rubén Escolano Garcia, Andrés Galeano Galeano Valiente, Maria Isabel Gallardo Vidal, Juliana García Cosmes, Maria Elena García López, Raquel García López, Belén Gómez Gómez, Maria Aurora Latorre Gálvez, Jose Luis Mijangos Rodriguez, Maria Luz Turégano Prieto

**Affiliations:** 1 Doctor Castroviejo Primary Care Health University Center. Northern Primary Care Health Directorate of the Community of Madrid, Madrid, Spain; 2 Medicine Department, Family Medicine and Primary Care Division, School of Medicine, Autonoma University of Madrid, C/ Arzobispo Morcillo, 2-4, 28049 Madrid, Spain; 3 Hospital La Paz Institute for Health Research (IdiPAZ), Madrid, Spain; 4 Health Sciences Department, “Santa Teresa de Jesús” Catholic University of Avila, Avila, Spain; 5 Valdelasfuentes Primary Care Health Center. Northern Primary Care Health Directorate of the Community of Madrid, Madrid, Spain; 6 Reina Victoria Primary Care Health University Center. Northern Primary Care Health Directorate of the Community of Madrid, Madrid, Spain; 7 Torrelaguna Primary Care Health Center. Northern Primary Care Health Directorate of the Community of Madrid, Madrid, Spain; 8 Miraflores Primary Care Health Center. Northern Primary Care Health Directorate of the Community of Madrid, Madrid, Spain; 9 Colmenar Viejo Norte Primary Care Health Center. Northern Primary Care Health Directorate of the Community of Madrid, Madrid, Spain; 10 Fuencarral Primary Care Health University Center. Northern Primary Care Health Directorate of the Community of Madrid, Madrid, Spain; 11 Barrio del Pilar Primary Care Health University Center. Northern Primary Care Health Directorate of the Community of Madrid, Madrid, Spain; 12 Research Network in Health Services and Chronic Diseases (REDISSEC), Madrid, Spain; 13 Family and Community Teaching Unit Norte. Primary Care Management. Madrid Health Service, Madrid, Spain; 14 Research support Unit. Primary Care Management. Madrid Health Service, Madrid, Spain; 15 Preventive Medicine and Public Health Department. Family Medicine and Primary Care Unit. School of Medicine, Autonoma University of Madrid, Madrid, Spain; 16 MEFAP Group (MEFAP Multicomponent physical activity program-Group, for its acronym in Spanish), Madrid, Spain

**Keywords:** Elderly, Physical activity, Exercise, Frailty, Randomised clinical trial, Primary health care

## Abstract

**Background:**

Physical activity may reverse frailty in the elderly, but we encounter barriers to the implementation of exercise programs in this population. Our main aim is to evaluate the effect of a multicomponent physical activity program, versus regular medical practice, on reverting pre-frailty status among the elderly, 12 months post-intervention.

**Methods:**

Randomized parallel group multicenter clinical trial located in primary care setting, among non-dependent and pre-frail patients > 70 years old, including 190 patients (95 intervention, 95 control group). Intervention: Multicomponent physical activity program (MEFAP, for its acronym in Spanish) with twelve 1.5 h-weekly sessions comprised of: 1. Informative session; 2. Exercises for improving aerobic resistance, muscle strength, propioception-balance and flexibility; and 3. Handing out of at-home exercise chart (twice/week). Main variable: pre-frailty according to the Fried phenotype. Secondary variables: sociodemographic, clinical and functional variables; exercise program adherence, patient satisfaction with the program and quality of life. We will perform an intention-to-treat analysis by comparing the retrogression from pre-frailty (1 or 2 Fried criteria) to robust status (0 Fried criteria) by the end of the intervention, 6 months and 12 months post-intervention. The accumulated incidence in each group will be calculated, as well as the relative risk (RR) and the number needed to treat (NNT) with their corresponding 95% confidence intervals. Protocol was approved by the Ethics Committee Hospital la Paz.

**Discussion:**

Within the context of regular clinical practice, our results will provide evidence regarding the effects of exercise interventions on frailty among pre-frail older adults, a key population given their significant potential for functional, physical, and mental health improvement.

**Trial registration:**

NCT03568084. Registered 26 June 2018. Date of enrollment of the first participant to the trial: July 2nd 2018.

## Background

Worldwide, Spain enjoys one of the highest life expectancies and population projections point to a continuing population ageing process [1].

The 2012 European region committee for The World Health Organization approved the “Strategy and action plan for healthy aging in Europe 2012-2020” [2]. Similarly, the European Union, within the framework program Horizon 2020, launched a program for “the cooperation for innovation in Europe regarding active and healthy ageing.” One of the program’s pillars of action is the prevention, screening, and early diagnosis of frailty and functional deficit [3].

In the context of healthy ageing, the older person stays healthy and remains independent longer thus reducing the family burden in terms of informal care. The opposite scenario is functional decline, i.e., frailty, defined as a decrease in the homeostatic reserve of the individual leading to increased vulnerability to stressors. Frailty is a reliable predictor of short, medium, and longterm adverse health events such as falls, multimorbidity, institutionalization, hospitalization, and even death [4]. Frailty prevention and control is currently an important public health challenge clearly associated to the hastened population ageing.

Frailty and pre-frailty are commonly found among the elderly. A recent systematic review estimates that about 10 and 44% of 65-year-olds or older are frail or pre-frail, respectively [5]. It is worth underlining that the frailty syndrome is reversible, that is, a frail individual can turn the clock back and become robust again as long as frailty is detected and treated at the onset [6]. Therefore, identifying and treating pre-frailty is effective in preventing disability and other adverse events, improving quality of life, and reducing care-related costs [7, 8]. Further, pre-frailty is the most appropriate time to implement interventions by the health care system as it is when the best patient response to these interventions can be expected [9].

Lowering frailty rates requires reducing inactivity, one of its main risk factors [10]. Both a 2012 and a 2014 meta-analysis studying fragile individuals showed that physical activity delays and even reverses frailty and disability [11, 12], improves cognitive status and promotes emotional well-being and socialization [13]. Specifically, multicomponent physical exercise programs comprised of muscle strength development, cardiovascular endurance, and joint mobility and balance, are the most effective interventions to delay disability and other adverse events as well as to maintain the highest degree of independence possible for each individual [14]. However, how different trials measure interventions varies which difficults results comparisons. Therefore, physical activity, insofar as it prevents and even reverses early stages of frailty, has the potential of averting falls, disability, and improving quality of life [15]. Not to be dismissed, mood enhancement and the socialization inherent to group exercise programs may also contribute to these benefits.

Furthermore, physical inactivity is also the primary cause of most chronic pathologies [16]. Therefore, it is of interest to analyze the evolution of the control parameters of these pathologies (especially those related to cardiovascular risk) usually monitored by primary care medical staff, from baseline through the follow-ups while performing physical exercise regularly for any substantial amount of time.

Other interventions such as increasing vitamin D levels, resolving an underlying anemia, or increasing protein intake have shown improvement in the elderly’s physical function, independently from regular exercise’s benefit, and should be implemented within the context of usual clinical practice [17].

In Spain, primary care, is not only the point of entry into the health system but it also provides accessibility to diverse strata of the population with low disease burden. Further, it offers continuity of care along with a comprehensive approach including disease prevention and health promotion [18]. Thus, primary care is ideally positioned to play a crucial role in this intervention program, through the maintenance and improvement of physical, cognitive, and social functioning among the elderly, as well as the promotion of individual self-care for as long as possible.

In addition, primary care is also the default for pre-frail individuals (close to 50% of all elderly) to receive care. Therefore, it is the ideal place for “exercise prescription,” for teaching the necessary skills, and, last but not least, for promoting self-care, participation, and individual empowerment [19]. Consequently, it is primary care’s responsibility to implement this type of program, upon documented evidence of its effectiveness in the context of clinical practice. Unfortunately, this evidence is scarce in Mediterranean countries [13, 20], especially with pre-fragile individuals [21, 22].

Long-term multifactorial interventions have been shown to be more cost-effective than regular clinical practice [9]. However, implementating these programs in clinical practice usually encounters significant barriers thus limiting their effectiveness. These barriers include, among others, inadequate planning and coordination among professionals, insufficient support at the service provider level, inadequate dedication of the personnel involved, and not active enough attitudes on the part of key participants [23, 24].

Adherence to exercise among previously sedentary frail or pre-frail elderly fails to reach even 50% [22], although it is somewhat higher for aerobic exercises such as walking or cycling versus strength exercises, and is also higher for group exercises versus proposed home exercises [25]. An exercise intervention pilot study in frail 65 year-olds and older performed by our team in the Primary Care Health Center Doctor Castroviejo (unpublished data), yielded a 28.3% adherence rate when defining adherence as participation in at least 70% of the sessions. Among the barriers limiting the recruitment and adherence to exercise programs, the more common included already getting enough exercise, not being motivated or ready and having poor health [26].

Currently in Spain, significant lines of research on exercise and healthy ageing are underway, although not of them has involved primary care [27, 28]. In fact and as far as we know, there have been few interventions performed exclusively with pre-frail individuals [21, 22], thus, such studies are needed to provide evidence generalizable to this population [9].

Based on this evidence, this project aims to create a multicomponent physical exercise program for pre-frail individuals. In contrast to previous ones, our project would be nestled within the structure and practice of primary care. We would examine its effectiveness as well as all and every aspect related to the implementation of such a program in the context of primary care.

## Aims

Main goal of the MEFAP-project is to evaluate the effect of a multicomponent physical activity program in the primary care setting, versus regular medical practice, in reversing pre-frailty status [4] 12 months post-intervention among patients over 70 years of age.

Secondary Objectives of the study are:

-To evaluate the effect of MEFAP versus regular medical practice at 0- and 6-months post-intervention regarding: pre-frailty status according to the Fried scale, quality of life, and clinical parameters such as blood pressure, body mass index (BMI), cognitive status, mood and chronic pain, and biological parameters such as metabolic and inflammatory markers.

- Assess adherence to MEFAP and participant satisfaction.

## Methods

### Design and setting

**Fig. 1:**
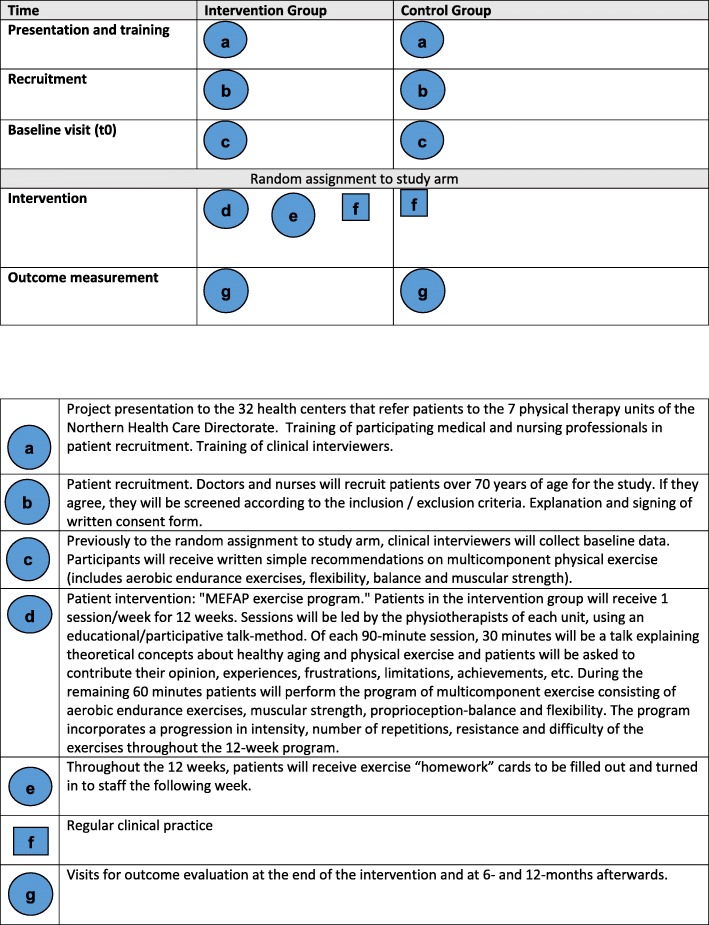
MEFAP Project Design**.** MEFAP: Multicomponent physical activity program (for its acronym in Spanish)

Pragmatic multicenter randomized clinical trial with parallel groups followed for 12 months. The setting is the primary health care of the Spanish national health system. Figure 1 shows the study design which was informed by SPIRIT guidelines, which will also guide the study reporting (Table 1).

**Table 1:** Schedule of enrollment, interventions, and assessments in MEFAP Project

TIMEPOINT	Enrolment	Allocation				End of Study
	*-t*_*1*_	0	*t*_*1 baseline*_	*Intervention*	*t*_*2*_ *6 –months post-intervention*	*t*_*3*_ *12–months post-intervention*
ENROLMENT						
Eligibility screen	X					
Informed consent	X					
Randomized Allocation		X				
INTERVENTION						
INTERVENTION GROUP: *Multicomponent Exercise Program (MEFAP)*				X		
*CONTROL GROUP: Regular Clinical Practice*				X		
ASSESSMENTS						
***Main outcome variables:***						
*● Frailty level (Fried Criteria)*	X		X		X	X
*Secondary outcome variables*						
*● Function-related variables: SPPB, risk of falls*			X		X	X
*● Quality of life*			X			X
*● Clinical Parameters:*						
✓ Blood pressure			X		X	X
✓ BMI			X		X	X
✓ Cognitive state (MEC30 Lobo)			X		X	X
✓ Mood (Yesavage test)			X		X	X
✓ Chronic-use drugs (last 2 weeks).			X		X	X
✓ Chronic pain						
*● Analytical Parameters (metabolic and inflammation).*					X	X
*● Program adherence and satisfaction level*			X			X
*Independent Variables*						
*● Sociodemographic*			X			
*● Comorbidity o diagnosis of ≥ 2 chronic disease*			X			
*● Fracture in previous 5 years*			X			

*MEFAP:* Multicomponent physical activity program (for its acronym in Spanish), *SPPB* short physical performance battery, *BMI* Body Mass Index, *MEC30 Lobo* Mini-mental Exam Cognitive (Translated and adapted to Spanish by Lobo et al. 2002 [33])

### Study participants

The study population is composed of non-dependent, pre-frail elderly patients over the age of 70 and attending the participating health centers.

### Inclusion criteria

Following the 2014 Consensus Document on the prevention of frailty and falls in the elderly patient [29] we will recruit individuals > 70 years of age, with a Barthel test score ≥ 90 and meeting at least one of the following two criteria: a score between 9 and 11 in the short physical performance battery (SPPB) [30] or FRAIL questionnaire with values 1 or 2 [31, 32]. At baseline (t1), pre-frail patients will be included (1 or 2 frailty criteria according to Fried: unintentional weight loss, exhaustion, weakness, slowness, and low physical activity) [4].

### Exclusion criteria

1. Inability to go to the Primary Care Health Center for any reason.
2. Moderate to severe cognitive impairment (MEC 30 Lobo ≤21) [33].
3. Severe pathology for which physical activity is contraindicated at the physician’s discretion including but not limited to: recent acute myocardial infarction (6 months), uncontrolled cardiac arrhythmia, severe cardiac valve disease, non-controlled hypertension (> 180/100), non-controlled/severe heart failure, severe respiratory insufficiency disease, and diabetes mellitus with acute decompensation/frequent hypoglycemia.

### Sample size

We calculated the sample size based on two data points: the published figure of 15% of pre-frail individuals that transition to robust status in the absence of an intervention [12] and our 35% estimated percentage of pre-frail elderly in the intervention group becoming robust again. Therefore, we expect a difference of 20% of favorable evolution (from pre-frail to robust) between both control and intervention groups. Considering a power of 80% and an alpha of 0.05, the sample size would be 146 patients (73 patients in the intervention group and 73 patients in the control group). Assuming an attrition of 30%, the final number needed to recruit is 190 patients (*n* = 95 in IG and *n* = 95 in CG).

### Recruitment

#### Primary Care Health Centers

All 32 Primary Care Health Centers (HC) in the Northern Primary Care Health Directorate of the Community of Madrid referring patients to the 7 physiotherapy units available in this catchment area will be offered to participate in the study. The principal investigator will contact and recruit each center personally.

#### Patients

Participating HC’s doctors and nurses will recruit patients > 70 years of age consecutively until reaching their HC’s patient quota (10–15 patients). The preselection in clinical consultation will be performed with the Barthel test, the SPPB, and the Frail questionnaire as an approach to the Fried frailty criteria, following the recommendations of the Consensus on the prevention of frailty and falls in the elderly, published in 2014 by the Spanish Ministry of Health, Social Services and Equality [29]. Eligible participants will at this time sign an informed consent and make an appointment to fill out the baseline survey (t1).

#### Randomization

Patients will be randomized into IG or CG after completion of baseline data collection (t1) in an automated process included in the Electronic Data Collection Notebook (eDCN).

##### Outcome

ᅟ

### Study variables

a) Main Outcome: Change in patient’s frailty level from pre-frail to robust. Frailty status is based on Fried criteria [4]: low gait when walking 4 m, grip strength with the dominant hand measured with a dynamometer, unintentional weight loss > 5% of body weight in the past year, low energy (CES-D) [34], and low physical activity (Yale scale) [35]. Pre-frailty is defined as meeting 1 or 2 Fried criteria and robust status is defined as meeting none (0) of Fried criteria.

b) Secondary outcome variables: Variables related to physical functioning: short physical performance battery (SPPB) [30], risk of falls (measured according to consensus proposal on the prevention of frailty and falls [29]. Quality of life: Measure with the EuroQol questionnaire 5D-5 L [36]. Adherence to the program and degree of satisfaction: Patients will be considered to have a good MEFAP adherence when they have attended at least 75% of the sessions**.** With regard to satisfaction, the dimensions of communication, professional attitude, technical competence and accessibility will be evaluated based on a questionnaire created for this purpose and measured with a Likert scale.

Clinical parameters: blood pressure, BMI, Cognitive state (MEC30 Lobo) [33] mood (Yesavage test) [37], chronic pain, and any chronic drugs consumed in the last two weeks. Analytical parameters: blood count, glucose and glycated hemoglobin, lipid profile, iron metabolism (iron, ferritin, transferrin), vitamin D, vitamin B12, folic acid, total proteins, and albumin and inflammation markers (ESR, CRP and fibrinogen).

c) Independent variables. Sociodemographic**:** age, sex, educational level, economic level (monthly family income in euros), main professional occupation. Clinical: Fracture in the previous five years (specify if hip fracture), Comorbidity or diagnosis of at least 2 diseases from the following list: Heart disease (arrhythmia, heart failure or ischemic heart disease), cerebrovascular disease, diabetes mellitus, chronic lung disease (asthma, COPD or respiratory failure), chronic kidney disease, cancer, and osteoarthritis / limiting arthritis (i.e., affecting activities of daily living).

#### Intervention

Intervention Group (IG): The intervention, MEFAP, consists of a multicomponent physical activity program that includes 1) informative talk; 2) aerobic endurance exercises (walking), muscle strength, proprioception-balance and flexibility; and 3) flashcards with guidelines of home exercises for at least twice that week. This is a 12 session-intervention to take place on a weekly basis in the physical therapy units with each session lasting for an hour and a half. To reduce interprofessional variability in the intervention, the participating physiotherapists will be trained to standardized the delivery of the intervention. Control group (CG): Participants assigned to this group will receive the usual clinical practice.

All participants will receive a document with simple physical activity recommendations as well as their report with the results from all the clinical and analytical tests after the baseline interview. Attrition will be minimized by contacting participants by telephone after missing just one intervention session.

### Data collection

Data will be collected using an electronic data collection notebook (eDCN) designed for the study. There are four visits planned: enrolment (-t1) baseline (t1), at 6 months (t2) and at 12 months (t3) of the end of the intervention. The flowchart according to SPIRIT guidelines can be seen in Table 1.

### Analysis strategy

Analyses will compare the intervention group (IG) and the control group (CG) and corresponding changes in frailty status, physical performance, cognitive level, mood, and chronic pain between baseline, and three post-intervention points (0, 6, and 12-month post-intervention). Baseline characteristics will be described according to qualitative or quantitative variables: description and analysis of the distribution of each variable, normality tests (Kolmogorov-Smirnov) and scatter charts. Participant attrition will be described in detail. A baseline comparison will be made between the groups: in terms of descriptive, outcome, and prognostic variables. The appropriate bivariate statistical tests will be used according to type of variable (qualitative or quantitative).

### Main analysis of effectiveness

Data will be analyzed using intention-to-treat analyses.

A positive event for the main outcome variable, intervention effectiveness, will be defined as the transition from pre-frailty (1 or 2 Fried criteria) to robust status (zero Fried criteria) during the study period. The accumulated incidence in each group, the relative risk (RR), the number-needed-to-treat (NNT) will be calculated, with their corresponding 95% confidence intervals. We will perform the analysis with the statistical programs SPSS v.24.

Outcome evaluation (changes in frailty) and statistical analyses will be conducted by skilled study staff blind to treatment allocation.

## Discussion

The main goal of health services, and primary care specifically, is the prevention of morbidity and mortality. To this end, the proposal of the Consensus on the prevention of frailty and falls [29] aims to introduce exercise into the regular routine of the elderly population to prevent and reduce functional decline. Within this framework, the study’s desirable outcome is to encourage the implementation of an exercise intervention tested in the community sphere so physical activity becomes a regular habit in the older adult population. A key characteristic of the study is its pragmatic design of primary care implementation, which not only allows to evaluate the program’s effectiveness but also its feasibility in conditions and settings similar to those where it would be implemented in the future. Therefore, it is also crucial to evaluate aspects such as accessibility to the intervention, adherence to the program, user satisfaction, and increase in quality of life.

The study presents the following limitations: First, there could be confounding effects if those assigned to the CG were to follow the intervention. In order to minimize this possibility and avoid attrition in the CG, these participants will be offered participation in a similar multicomponent exercise program at the end of the study period. Second, we expect a lower participation rate among individuals whose Primary Care Health Center is further away or the corresponding physical therapy center is not easily accessible by private or public transport. This information will become an important data point as social and health services must be highly accessible to the user. Thus, we propose to analyze the relationship between user accessibility (in time, distance and comfort) and adherence to program.

Third, participants will not be blind to group assignment due to the trial’s design. However, outcome evaluation will be conducted by skilled interviewers that are blinded for treatment allocation. The statistician conducting the analysis will neither know to which study group a given patient has been assigned.

One of the main strengths of the study is its pragmatic design placing the program implemention within primary health care, that is, in similar conditions to real-world implementation. This, in turn, may help the prescription of exercise become a therapeutic tool within the comprehensive care of the elderly patient.

Here we propose a multicomponent exercise intervention implemented at the primary care level based on healthcare professionals´ solid commitment to pursue the program participation and socialization of individuals.

Based on our knowledge of the characteristics of primary care in the Community of Madrid, the program’s successful implementation is feasible and, furthermore, we expect higher levels of adherence than those reported previously [23]. Favorable results in this endeavor would encourage us to support the implementation of periodic programs for the improvement of the quality of life and physical and mental function of the population served in primary care.

## Abbreviations

BMI: Body Mass Index
CG: Control group
eDCN: Electronic Data Collection Notebook
IG: Intervention group
MEFAP: Multicomponent physical activity program (for its acronym in Spanish)
NNT: Number needed to treat
RR: Relative Ratio
SPPB: Short Physical Performance Battery
